# The engagement of older people living with chronic lung disease in a peer support community‐based exercise programme: A qualitative study

**DOI:** 10.1111/hex.13847

**Published:** 2023-08-12

**Authors:** Rebekkah Middleton, Christine Metusela, Kelly Marriott‐Statham, Caleb Ferguson, Patricia M. Davidson

**Affiliations:** ^1^ School of Nursing University of Wollongong Wollongong New South Wales Australia

**Keywords:** breathlessness, chronic lung disease, community‐based programmes, peer support, pulmonary rehabilitation

## Abstract

**Background:**

Chronic lung disease is a common and complex condition. Pulmonary rehabilitation programmes—either hospital‐based or in the community are recommended in evidence‐based clinical practice guidelines.

**Aim:**

To explore the experience of older people with chronic lung disease involved in a peer support community‐based exercise maintenance programme.

**Design and Method:**

Participants were a part of the *Lungs in Action* programme run in a local community leisure centre through Lung Foundation Australia. All the programme participants (*n* = 25) were invited by an independent person through email and/or letter to participate in the study and provided with a participant information and consent form. Participants who returned consent forms were scheduled for group interviews. Participants were recruited over a 2‐week period between 30 August and 13 September 2022. We conducted qualitative group interviews using a semi‐structured interview guide to explore the experiences of older people living with chronic lung disease. Data were analysed using reflexive thematic analysis.

**Results:**

A total of 14 participants (eight female and six male) aged between 64 and 86 years were interviewed. Three themes emerged from the data: *motivation, authentic social engagement, and sustainable achievement*. Motivation stemmed from the participants' perceived health benefits, and from the trainers' motivation and encouragement. Participants discussed how sharing experiences created an environment of trust and understanding, fun and friendship. Social engagement and creating authentic relationships were key aspects raised by participants. Feeling more confident in themselves and being able to accomplish physical tasks, making activities of daily living more manageable featured highly in participants' responses.

**Discussion and Conclusion:**

Community‐based peer support exercise groups enable environments for people with chronic lung disease to maintain physical fitness, and to connect with others to form friendships and have fun.

## 1 INTRODUCTION

Globally, advanced ageing and multimorbidity are key contributors to increased health system burden, and socioeconomic cost.^1^, ^2^, ^3^, ^4^ Chronic lung disease represents a growing burden internationally, with 4.1 million deaths annually.^1^, ^5^, ^6^ In Australia, one in every three people is affected by chronic lung disease,^7^, ^8^ and most are aged over 70 years, contributing to 8% of the total burden of disease.^9^

When chronic lung disease is diagnosed by a respiratory specialist, over 90% of people are referred to a community or hospital‐based pulmonary rehabilitation exercise and education programme.^10^ These evidence‐based care pathways are cornerstone treatments for people with chronic respiratory disease,^11^ and ensure improved exercise capacity, health‐related quality of life and reduced hospital admission.^12^, ^13^ Referral to these programmes is a first step to equipping individuals with the skills needed to exercise safely and manage breathlessness, reducing the frequency of acute exacerbations, and thereby helping people effectively self‐manage, remain well and avoid hospitalisation.^12^ Exercise tolerance is a key element needing to be maintained following pulmonary rehabilitation to maintain the benefits achieved. This is a priority for all pulmonary rehabilitation programmes globally.^14^

Although structured exercise programmes do not differentially impact exercise adherence,^15^ it is evident there are several other benefits for older people, such as peer support,^16^ fostering friendships^17^ and minimising loneliness^18^ and social isolation.^19^ Community recreational activities and programmes support ‘healthy ageing’,^20^, ^21^ and enable older people to maintain cognition, improve confidence, self‐belief, and quality of life,^22^ along with benefiting socially from being engaged in such community programmes.^23^, ^24^, ^25^ Traditionally, community‐based exercise programmes for older people have primarily focused on promoting physical function, stability and/or independence and decreasing frailty, due to physical activity participation declining with age.^26^

By empowering participants to take responsibility for their own health and well‐being and engage with others, a fostering of an internal locus of control and an increased ability to manage change occurs.^27^ Shifting the focus to the person involved, self‐management and self‐determination are encouraged, bringing a positive understanding of health and empowerment to the individual. This can promote connection and peer support with others in a positive and supportive environment with people who have a shared lived experience of chronic disease.^22^ Many older people choose to participate in an exercise programme if they can do it with others in a social manner with the possibility of forming and maintaining positive and supportive friendships.^25^ Formal interventions such as *Lungs in Action* offer opportunities for participants to share a unique type of social support with others who share characteristics,^28^ in this case chronic lung disease. Shared lived experiences in formal programmes enables peer support in helping each other to manage the same condition, potentially offering a sense of connectedness and purpose.^29^

In Australia, people with chronic lung disease, following pulmonary rehabilitation, are regularly referred to as *Lungs in Action*.^30^ *Lungs in Action* is conducted throughout Australia in approximately 35 locations. It is conducted to maintain the health benefits initially achieved in acute pulmonary rehabilitation programmes for people with chronic lung disease, building skills to live well with chronic lung disease in the community. The aims of *Lungs in Action* are to improve fitness, independence and connection to the community.^30^ *Lungs in Action* is a community‐based programme led by trained professionals leading tailored exercises twice a week for participants, inclusive of resistance training and cardio (walking).^30^ The programme enables opportunities to exercise safely and to meet others with a lived experience of chronic lung disease in a supportive, understanding community. This research explored a group of older people (over 60 years) with chronic lung disease who had participated in *Lungs in Action* for up to 9 years together. Older people commonly desire social engagement and seek opportunities to be involved with others.^31^ There is a need to explore the social engagement needs and participation of people living with chronic lung disease. Given the impact of social isolation and lockdowns due to the COVID‐19 pandemic, this is of particular importance, as evidenced in the United Nations' Decade of Healthy Ageing 2021–2030 document.^2^

### 1.1 Aim

To explore the experience of older people with chronic lung disease involved in a peer support community‐based exercise maintenance programme.

## 2 DESIGN AND METHODS

### 2.1 Setting

Participants in the study were all participants of the *Lungs in Action* community programme run through Lung Foundation Australia. The exercise programme in this research is located in Wollongong, 80 km south of Sydney, New South Wales, Australia.

### 2.2 Study design

The research study methodology is informed by a critical ontological perspective where multiple realities are accepted. The ontological perspective this research study took was a person‐centred^32^ perspective where the intent of the research is to explore the reality that exists within the context where the research is undertaken for the people involved. This research design sought to better understand the experience of the participants, gaining insight into key aspects of engaging in a peer support community.

To achieve understanding, we conducted qualitative group interviews with participants of the *Lungs in Action* community exercise programme. Group interviews are evidenced to provide a synergistic setting to gather insight into cultural and community norms.^33^ They provide an environment where participants are influencing and influenced by others, like in real‐life settings.^34^ The interview guide is available in Supporting Information: Appendix S1.

### 2.3 Participant recruitment

Recruitment was facilitated through the assistance of the community leisure centre where the *Lungs in Action* programme is delivered. All programme participants (*n* = 25) were invited by an independent person through email and/or letter to participate in the study and given a participant information and consent form. Participants who returned consent forms were scheduled for group interviews by researcher K. M. S. who had no pre‐existing relationship with any of the participants or the *Lungs in Action* programme. Participants were recruited over a 2‐week period between 30 August and 13 September 2022. Inclusion criteria were all *Lungs in Action* programme participants. Exclusion criteria were anyone not enroled in the *Lungs in Action* programme.

One of the researchers (R. M.) is also a personal trainer who provides exercise instruction and support for the *Lungs in Action* participants. Due to this pre‐existing relationship, she had nothing to do with recruitment or data collection.

### 2.4 Data collection

We developed a semi‐structured interview guide to explore the experiences of older people living with chronic lung disease being involved in a community peer support exercise programme. Questions included how participants were introduced to the programme and the perceived benefits of the programme. Participants were asked to discuss their perspectives and experiences of being involved in the *Lungs in Action* programme, including what is important to them about being involved and the key benefit for them personally.

Interview guide development was informed by the literature and reviewed by an expert panel, including study investigators. Both group interviews were conducted face‐to‐face by an experienced qualitative researcher (K. M. S.) at the centre where the programme is facilitated. The interviews were an average of 58 min (SD = 0.5445) in duration. Interviews were audio‐recorded and transcribed verbatim, using a third‐party professional transcription service. Transcripts were checked for accuracy by checking against the audio recording (K. M. S.) and deidentified with participants being assigned labels.

### 2.5 Analysis

Reflexive thematic analysis was conducted to interpret the experiences and perspectives of participants. Reflexive thematic analysis is an inductive, data‐driven, iterative approach enabling patterns and meanings to be captured from qualitative data.^33^, ^35^ This ensures the themes identified best reflect participant views without fitting them into a preconceived coding framework.^34^ Four members of the research team (R. M., C. M., K. M. S., P. D.) each individually coded both group interview transcripts to identify categories within the data before consulting as a group to check and agree on the initial codes and consider differences in interpretation. To develop a richer more nuanced reading of the data, three of the team (R. M., C. M., K. M. S.) further reviewed and refined the coding frame to include emerging new codes and suggest overarching themes. The final thematic structure was agreed by the research team to clearly and comprehensively describe our analysis and we agreed saturation of codes had been achieved.

Throughout the process, we engaged collaboratively and reflexively, aware that our experiences and sociocultural backgrounds shaped our interpretation.^33^, ^35^, ^36^ The four members of the research team robustly discussed each stage of analysis using Braun and Clarke's method of reflexive thematic analysis^35^ until coming to consensus with regard to the final themes. Each member was reflexive in their approach, aware of subjectivity and potential influences. This encouraged a deep sustained data immersion and reflection.^35^

This study was approved by the University of Wollongong's Human Research Ethics Committee (2022/248). The COREQ checklist on qualitative reporting^37^ is provided in Supporting Information: File S1.

## 3 RESULTS

Data were collected from a total of 14 participants ranging in age from 64 to 86 years. Eight were female and six were male. There were eight participants in group interview 1 and six in the second.

**Figure 1:**
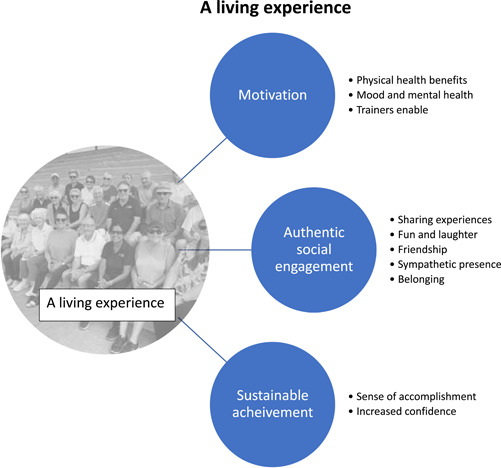
A living experience.

We identified three themes describing participants' perceptions and experiences of the peer support group: *motivation*; *authentic social engagement*; and *sustainable achievement*. An overarching premise encompassing all three themes is *living experience*. Figure 1 illustrates the themes and subthemes. Each theme and related subtheme are described below and illustrated with exemplar quotations. Quotes are attributed to participants using numbers, for example, P1 and the group interview, for example, GI1.

### 3.1 Motivation

Study participants perceived the *physical health benefits* of the exercise programme were a key motivator to continue with the programme, with comments such as ‘they explain to you with that rehab thing that if you exercise and get your large muscles stronger, then your recovery—the oxygen you have you can do more with it… and once you get that in your brain, it's a pretty good motivator to do it’ (P2, GI2).

Participants felt the exercise programme was helping to improve their health and well‐being; ‘Well, I think my health is improving and I'm getting a bit of fitness back, and I'm breathing a bit easier because I was really breathless at one stage. So that's really helping me’ (P7, GI1). Several participants were motivated to get fitter to be able to play with their grandchildren. Others mentioned the programme helped keep them out of hospital; ‘Well, the knowledge that it's important to do it. It's definitely a motivator. I don't want to go back into hospital if I can help it’ (P2, GI2). Some were determined not to be a burden on their family; ‘I don't want to be a burden to my family’ (P1, GI2) and thus were motivated to continue with the programme.

Many participants also perceived the programme helped improve *mood and mental health*. Improvements in their physical health appeared to have a flow on effect to their mental health. As one participant said ‘it's a positive thing for the mind to know that you can do a bit better, that you can do some things. You can go for a walk. And the thing that I've learnt—it's all right to be out of breath. It's Ok’ (P3, GI2). Participants spoke about the exercise programme in terms of giving them ‘a reason to get out of bed’ (P2, GI1), lifting their mood and giving them energy:

It does lift your mood…You force yourself to come because you know when you come out of here, your endorphins are all flying. (P2, GI2)

The other thing about coming to the group is you might come, and you've got no motivation, or no energy and you start doing the exercise and that and by the time you get to the end, you feel good. You feel alive. You walk out of here with a spring in your walk… it's motivational… when you're at home and you're feeling pain, you reflect back to what's been done here and you think, ‘Yes, they felt good, that did help’. (P7, GI1)

Participants identified the *trainers enabled* and motivated them, taking an interest in them as people and giving them individual attention. This was expressed through comments such as ‘And I think the other thing you need to give credit to is the trainers because they do take an interest in us and they just pull you aside and say, “What's happening today?” and they perhaps give you some suggestions what to do and what might help you. And generally, it's very helpful. It's been very helpful’ (P2, GI2).

Participants identified that they were motivated to continue in the programme because of their trainers who encouraged and inspired them; ‘Well, we love our trainers. We think they're very good and they're very valuable and they're inspirational to us. They motivate us. They help us. We couldn't do without them either’ (P7, GI1).

Participants spoke of the commitment of trainers during Covid, who thought up innovative ways to keep the programme going in some capacity: ‘We met at the park… and we exercised there between the trees’ (P2, GI1), ‘and we did it even online’ (P3, GI1).

Participants also told of the social aspect of the group being a motivator and believed without the group and their trainers, they may not have kept up their exercises:

Yeah, the social aspect, that's pretty important because I'm sure I wouldn't do it if this wasn't—If I didn't have to come out and this wasn't a group, I wouldn't do it. And that would be bad for me. (P2, GI2)

But if it wasn't for the group that we have, I probably wouldn't bother too much. That's the thing that draws you in. You know the people and our trainers have been great. (P5, GI1)

### 3.2 Authentic social engagement

A key aspect raised by all participants was social engagement and creating authentic relationships. Participants discussed how *sharing experiences* created an environment of trust and understanding. ‘We've all got a common purpose too. I think that melds us together a bit, all the conditions and empathise with each other’ (P2, GI2), ‘When we're here you know we're all in the same boat, we all understand’ (P1, GI2). This included sharing things related to their lung disease, ‘And we discuss our medications amongst ourselves sometimes too, see what other people are taking, what you are, see whether perhaps there's something that can help you more also different therapies and stuff going, and pass that around’ (P2, GI1), and learning generally, ‘You can always learn something from someone. You're never too old to learn’ (P1, GI1). Sharing in authentic interactions was a key aspect participants mentioned they missed during COVID‐19 (when the programme could not run due to risk), ‘So for me, I have missed that very, very much, and I'd be sitting there thinking, “I feel so down”, and you miss that contact’ (P5, GI1).

Participants talked about how *fun and laughter* were a large part of authentically engaging in the *Lungs in Action* programme. Being together and sharing ‘all the giggles that we have’ (P5, GI1), ‘the humour’ (P4, GI1), and noting ‘we do tease each other quite a bit’ (P2, GI1). The participants joked together about having a nickname for the group—‘Well our nickname is—its *Lungs in Action*, we call it “tongues in action”’ (P4, GI1).

Participants spoke fondly of social events such as ‘At the end of the year, we have a lunch at one of the clubs here and [trainers] give out awards for all of us, silly things. But it's a fun day. They come out with these terrific—what he says about us, award for something like this. They call me Mr Tin Legs because I've got two artificial knees. So they gave me a tin hat at one stage’ (P4, GI1). Another participant (P5, GI1) laughed as she talked about when ‘We all did Nutbush. We did. We all learned to do it. I buggered it up every time’. P2, GI2 added ‘And then we had [group member]. We had him trying to teach us the Greek dancing too, didn't we?’.

These experiences of *fun and laughter* impacted participants beyond the time spent in the class on the day, with some noting ‘It's a bit of fun, so when you leave here, I'm not sad. You feel like going home and doing something’ (P4, GI2), and P5, GI2 adding ‘Yes. I do more when I get home from here than if I'm home all day doing—you know’.

*Friendship* engenders fun and laughter and vice versa.^29^ Participants in this study focused on the importance of engaging in the *Lungs in Action* programme to ‘Make friendships and get to know other people and their way of lives and about their families’ (P7, GI1). A shared health condition provided common ground initially, enabling an immediate connection which led to friendship, ‘All the friendships you form and then we've had some organised outings and things like that, and it's lovely to talk to other people with similar problems and we tend to support each other’ (P1, GI1). ‘And we didn't know each other. We meet through here, and we're going all right. She's a really good cook. She invited me to lunch or whatever, I never say no’ (P5, GI2).

Some people noted deeper levels of friendship that had emerged since becoming part of the *Lungs in Action* programme, ‘I know with [group member], we've become pretty good friends. And I'll ring her up and say, “Are you OK?” whatever, and vice versa, and that happens, yeah’ (P5, GI1), (P7, GI1) ‘Yeah, we check on each other too, don't we? You miss a class; you see if you're OK. And a few of us go out for lunch every so often’, (P8, GI1) ‘We've been bowling’, (P1, GI1) ‘Yeah, we've been ten‐pin bowling. We had to have the things up so they wouldn't go in the gutter (laughs)’.

A *sympathetic presence* was evident as participants talked, one that demonstrated being seen, caring and cared for, and one of genuine interest, *‘*And I think the support we give each other, it's wonderful. If we think of something that would help somebody, we say, ‘Well, that might help you because I've had that and I've done that’ (P2, GI1). This included with and from the trainers, ‘The good thing is that we've had [trainers] from the beginning, so they have learned to know each person and each person's problems, and they really remember each person as a person, and they know, and they do cater for us individually. If one day, you feel not so well, they can tell. They can ask you to do something not so strenuous. But they really know us, I think. So good to have the same people’ (P3, GI2).

Participants described how they felt like a family, not alone—a real sense of *Belonging*. This was stated clearly in phrases such as ‘I'm really happy too, happy because I have like a family here, someone to talk to and you say hello’ (P5, GI2) and ‘It's like a family to me, to me anyway’ (P4, GI2). This ensured participants were ‘not so lonely. You think, “Oh yes, there's someone there to help me get through this”’ (P7, GI1) and felt they were ‘not alone [in their disease]. You suffer alone, so you're not suffering alone anymore’ (P4, GI2).

*Authentic Social Engagement* encompassed an array of elements to the participants, perhaps summarised best by (P7, GI1) who stated ‘I like the friendships, the conversation, the laughing, just the exercise, you're doing things. Time goes fast when you're talking and having a good time with your friends and that. I like the trainers. The trainers are good value’.

### 3.3 Sustainable achievement

Participants were asked to outline key benefits for them as a part of being involved in the *Lungs in Action* programme. Feeling more confident in themselves and being able to accomplish physical tasks, making activities of daily living more manageable featured highly in participants responses, leading to the theme of *Sustainable Achievement*. A *sense of accomplishment* was reflected in phrases such as, ‘It does feel so good when you do it. It makes you feel a million dollars, “I did that”. Yeah, it's brilliant’ (P5, GI1). Accomplishment was reported in terms of being able to do things which had not been achieved for some years prior, such as (P2, GI1) who stated ‘I've had both knees and both hips done, and I can finally do up my shoes and get my shoes on now without assistance… So that for me is a big thing because when it takes half an hour to get your shoes on before you go out, it's a bit of a problem’, and (P3, GI2) who reflected ‘For me, it's also the—it's up here knowing that you can do more things because your muscles are a bit stronger and you can—you feel a bit better because you can play with your grandchildren, get up off the floor. If you just sit and you do nothing, it's not that my lungs are going to get better, but the little bit that I've got, I can use’. Participants reported by strengthening their bodies, physical tasks at home were easier, ‘For me, that's fantastic because now I can do a few things at home’ (P5, GI2), and (P2, GI1) ‘But it's good to be able to do it because that means then if you're squatting somewhere, you can get up’.

Supporting participants to increase and improve tolerance for exercise led to *confidence*, and this was reported as important to participants, ‘Confidence is a big thing’ (P2, GI2). This was often linked to self‐esteem, ‘I think it improves your self‐esteem because you can see what you achieve and you realise that you can do it, and we wouldn't have known about it if I hadn't have come. It makes an amazing difference, actually’ (P5, GI2). An increased level of confidence contributed to a positive approach to life and was attributed to being a part of the programme, ‘I think for me is the positivity of life to know that I can do—I can live a better life by coming and exercising, and that I can do things that probably I would give up and think, “Oh, I can't do anything”. But I can. I can do a lot of things that are still good, and so it's really a positive thing. The key thing for me is positivity for my life, knowing that I can—because I can, because my muscles work better’ (P3, GI2).

## 4 DISCUSSION

When data is viewed through the lens of a person‐centred ontological perspective, participant voices tell a story of their reality. Participants' voices detailed their ‘living experience’ of the Lungs in Action programme, enabling three key themes were to be identified: including (1) motivation; (2) authentic social engagement; and (3) sustainable achievement. These findings are comparable to previous research that has explored the impact of similar programmes, such as the ‘Breathe Easy’ voluntary group network,^38^ and other peer support programmes for ongoing community‐led chronic disease management.

### 4.1 Peer support programmes help support motivation and the development of self‐efficacy

Self‐efficacy and motivation are important programme outcomes, often attributed to peer support, as elucidated in our study. However, a recent systematic review of peer support for people with chronic conditions revealed this study outcome was only assessed in approximately 45% of the 31 studies included.^28^ Peer support is considered a complex community‐based social intervention, underpinned by both social identity theory^39^ and social comparison theory.^40^ Community and social group membership can help to inspire a sense of belonging and can provide a behavioural framework through a set of social norms and conventions. Further, peer support may be applied by participants to compare themselves to others and used to validate their experiences and judge comparisons to create a sense of hope, as part of their self‐management and recovery. Hope and ambition are important to consider when setting personal goals.^28^ These goals varied within the programme, although included walking, sharing meals and social activities such as ten‐pin bowling and dancing.

### 4.2 Facilitating social connection and friendship

The *Lungs in Action* programme helped facilitate connection and friendship. This is supported by comparable evidence suggesting peer support programmes can help reduce social isolation, which can be associated with shame, rejection and stigma when living with chronic disease, including lung disease.^29^ Shared lived experience and shared chronic conditions may contribute to fostering bonds and connections between peers. This may enable them to make meaning of their own chronic disease and hence support coping and adjustment that may translate to improvements in overall self‐care and self‐management.^29^

### 4.3 Instilling confidence through shared experience

Research conducted by Hashem and Merritt^41^ found that groups such as ‘Breathe Easy’ create the opportunity for shared experience and provide an invaluable peer support network to facilitate the instillation of confidence. Peer support is considered a core component of programmes to support self‐management for patients with chronic disease. A recent study by Aboumatar et al.^42^ examined the addition of peer support to formalised and structured healthcare support programmes for people living with chronic obstructive pulmonary disease (COPD). They found that patients who received peer support in addition to healthcare professional support for COPD had fewer COPD‐related acute care events at 6 months, yet this failed to translate to improved health‐related quality of life.^42^

### 4.4 Provision of a sustainable transition pathway

Community‐led and community‐based peer support programmes such as *Lungs in Action* can provide a logical and sustainable transition pathway from more formal, structured healthcare orientated chronic care models (such as pulmonary rehab) or used as an adjunct to existing care structures. The *Lungs in Action* programme challenges the medicalised paradigm of chronic care for those living with respiratory and pulmonary conditions. Important aspects of this programme include the emotional and social components that do not generate traditional biomedical hierarchies of power or dependency.^40^ Emotional support is increasingly frequently lacking in traditional biomedical models, and it is clear these peer support community‐based models can provide an ongoing and sustainable framework for emotional and social support.

This study adds new understanding of the critical role of consumer‐led, community‐based peer support groups such as *Lungs in Action*. This presents a sustainable transition model of community social and emotional care for those living with pulmonary disease. Further, formalising the transition from organised and structured health‐system focused programmes such as pulmonary rehabilitation to community‐based *Lungs in Action* programmes should be considered.

## 5 LIMITATIONS

This study has provided rich and useful insights into the experiences and perspectives of participants of the *Lungs in Action* programme. However, this study is not without limitations. First, the sample was small although data saturation was achieved after the conduct of two focus groups. Further, given the focused sampling strategy, these results have limited transferability and may not be relevant across other chronic disease contexts. Future research is warranted to quantitatively evaluate the impact of community‐based programmes on health and well‐being outcomes to inform evidence‐based practices and policies supporting individuals living with chronic lung disease.

## 6 CONCLUSION

This study highlights the importance of these programmes in facilitating shared experiences of living with lung diseases, through peer support that offered emotional and social connection, fitness, friendship and fun. These are important factors to instil confidence, aide the development of self‐efficacy and may contribute to improved outcomes. The results of this study have important implications for healthcare providers, policymakers, and public health organisations, as they suggest the need to prioritise community‐based peer support programmes as a critical component of care for individuals living with chronic lung disease.

## AUTHOR CONTRIBUTIONS

**Rebekkah Middleton**: Conceptualisation; methodology; formal analysis; resources, writing—original draft, writing—review and editing, supervision, project administration, funding acquisition. **Christine Metusela**: Formal analysis; resources; writing—original draft. **Kelly Marriott‐Statham**: Formal analysis; data curation. **Caleb Ferguson:** Writing—original draft. **Patricia M. Davidson**: Formal analysis; supervision.

## CONFLICT OF INTEREST STATEMENT

The authors declare no conflict of interest.

## ETHICS STATEMENT

This study was approved by the University of Wollongong Australia Human Research Ethics Committee (2022/248). All related materials including participant information sheet and consent were approved by the Committee. The conception for the study involved discussions personally and online meetings with 10 people with chronic lung disease and two involved in healthcare.

## Supporting information

Supporting information.

Supporting information.

## Data Availability

The data that support the findings of this study are available from the corresponding author upon reasonable request. The complete thematic table with quotations is available on request.
