# Supplementary material for: The engagement of older people living with chronic lung disease in a peer support community‐based exercise programme: A qualitative study
Source: Health Expect. 2023 Aug 12;26(6):2409–17. doi: 10.1111/hex.13847 (PMC10632631; doi:10.1111/hex.13847)
Supplement: Supplementary file 1 — Supporting information. [file HEX-26--s002.docx]

Consolidated criteria for reporting qualitative studies (COREQ): 32-item checklist

| **No** | **Item** | **Guide questions/description** | **Completed by researchers** |
| --- | --- | --- | --- |
| **Domain 1: Research team and reflexivity** | | | |
| Personal Characteristics | | | |
| 1. | Interviewer/facilitator | Which author/s conducted the interview or focus group? | Marriott-Statham conducted the interviews. |
| 2. | Credentials | What were the researcher's credentials? *E.g. PhD, MD* | Middleton – PhD, MN(Res), BN, Dip.App.Sc.(Nursing), Grad Cert Emergency Nursing, Grad Cert Management, RN  Metusela – PhD  Marriott-Statham – PhDc, BN, RN  Ferguson – PhD, M Health Leadership, BSc (Nursing), RN  Davidson – PhD, BA Med, RN |
| 3. | Occupation | What was their occupation at the time of the study? | Middleton – RN, Senior Lecturer  Metusela – Senior Lecturer  Marriott-Statham – RN, Lecturer  Ferguson – Associate Professor  Davidson – Professor |
| 4. | Gender | Was the researcher male or female? | Middleton – Female  Metusela – Female  Marriott-Statham – Female  Ferguson – Male  Davidson – Female |
| 5. | Experience and training | What experience or training did the researcher have? | Middleton – PhD, MN(Res), Previous research/ethics/ implementation/write-up experience  Metusela – PhD, Previous research/ethics/ implementation/write-up experience  Marriott-Statham – Previous research/ethics/ implementation/write-up experience  Ferguson – PhD, extensive research/ethics/ implementation/write-up experience  Davidson – PhD, extensive research/ethics/ implementation/write-up experience |
| Relationship with participants | | | |
| 6. | Relationship established | Was a relationship established prior to study commencement? | Middleton is also a personal trainer who trains the participants. She had nothing to do with recruitment or data collection. All data was de-identified prior to analysis. |
| 7. | Participant knowledge of the interviewer | What did the participants know about the researcher? e*.g. personal goals, reasons for doing the research* | Participants knew the position that each researcher held.  Participants knew the reasons the researchers wanted to undertake the research, as outlined in PIS. |
| 8. | Interviewer characteristics | What characteristics were reported about the interviewer/facilitator? e.g. *Bias, assumptions, reasons and interests in the research topic* | Information about the interviewers is set out under subheading ‘data collection’. |
| **Domain 2: study design** | | | |
| Theoretical framework | | | |
| 9. | Methodological orientation and Theory | What methodological orientation was stated to underpin the study? *e.g. grounded theory, discourse analysis, ethnography, phenomenology, content analysis* | Qualitative descriptive approach.  Thematic analysis. |
| Participant selection | | | |
| 10. | Sampling | How were participants selected? *e.g. purposive, convenience, consecutive, snowball* | Purposive. |
| 11. | Method of approach | How were participants approached? e*.g. face-to-face, telephone, mail, email* | Participants were invited by an independent person through email and/or letter to participate in the study. |
| 12. | Sample size | How many participants were in the study? | 14 |
| 13. | Non-participation | How many people refused to participate or dropped out? Reasons? | No one refused or dropped out. Others that were invited did not respond. |
| Setting | | | |
| 14. | Setting of data collection | Where was the data collected? e*.g. home, clinic, workplace* | Leisure centre where Lungs in Action program runs. |
| 15. | Presence of non-participants | Was anyone else present besides the participants and researchers? | No. |
| 16. | Description of sample | What are the important characteristics of the sample? *e.g. demographic data, date* | 6 male, 8 female.  Aged between 64 and 84 years |
| Data collection | | | |
| 17. | Interview guide | Were questions, prompts, guides provided by the authors? Was it pilot tested? | Yes – semi-structured questions and additional prompts if required.  Not pilot tested. |
| 18. | Repeat interviews | Were repeat interviews carried out? If yes, how many? | No. |
| 19. | Audio/visual recording | Did the research use audio or visual recording to collect the data? | Audio recording. |
| 20. | Field notes | Were field notes made during and/or after the interview or focus group? | Made during interview. |
| 21. | Duration | What was the duration of the interviews or focus group? | 57 minutes and 29 seconds – 58 minutes and 5 seconds |
| 22. | Data saturation | Was data saturation discussed? | Yes. |
| 23. | Transcripts returned | Were transcripts returned to participants for comment and/or correction? | No. |
| **Domain 3: analysis and findings** | | | |
| Data analysis | | | |
| 24. | Number of data coders | How many data coders coded the data? | 4 |
| 25. | Description of the coding tree | Did authors provide a description of the coding tree? | Yes. |
| 26. | Derivation of themes | Were themes identified in advance or derived from the data? | Derived from data. |
| 27. | Software | What software, if applicable, was used to manage the data? | None. |
| 28. | Participant checking | Did participants provide feedback on the findings? | No. |
| Reporting | | | |
| 29. | Quotations presented | Were participant quotations presented to illustrate the themes / findings? Was each quotation identified? e*.g. participant number* | Yes. |
| 30. | Data and findings consistent | Was there consistency between the data presented and the findings? | Yes. |
| 31. | Clarity of major themes | Were major themes clearly presented in the findings? | Yes. |
| 32. | Clarity of minor themes | Is there a description of diverse cases or discussion of minor themes? | Yes. |
