# Supplementary material for: The engagement of older people living with chronic lung disease in a peer support community‐based exercise programme: A qualitative study
Source: Health Expect. 2023 Aug 12;26(6):2409–17. doi: 10.1111/hex.13847 (PMC10632631; doi:10.1111/hex.13847)
Supplement: Supplementary file 2 — Supporting information. [file HEX-26--s001.docx]

**Group interview guide**

**Project Title:
Experiences of a peer support exercise program for people with chronic lung disease (PeSEP)**

| Group interview guide |
| --- |
| 1. Tell me about your experience of being involved in this peer support community exercise program? 2. What were the reasons you joined this program? 3. What is important to you about being a part of this program? 4. What did you miss about this program when you were isolated in the lockdowns? 5. Is social engagement with others in the program important to you? 6. What is the key thing you benefit from participating in the program? |
